# The comfort and safety of a novel rolling mechanical indentation device for the measurement of lumbar trunk stiffness in young adults

**DOI:** 10.1186/s12998-017-0153-z

**Published:** 2017-08-03

**Authors:** Benjamin T. Brown, Alexandra Blacke, Vanessa Carroll, Petra L. Graham, Greg Kawchuk, Aron Downie, Michael Swain

**Affiliations:** 1 Department of Chiropractic, Macquarie University, Balaclava Rd, North Ryde, NSW 2109 Australia; 2 Department of Statistics, Macquarie University, Balaclava Rd, North Ryde, 2109 NSW Australia; 3 Department of Physical Therapy, University of Alberta, Edmonton, Canada

**Keywords:** Low back pain, Spine, Trunk, Stiffness, Adult

## Abstract

**Background:**

The measurement of Posterior-Anterior (P-A) spinal stiffness is a common component of the physical examination of patients presenting with spinal disorders. The aim of this assessment is to provoke pain and/or to determine the degree of resistance or compliance of these structures and the associated soft-tissues to loading. This information, combined with other patient-specific history and examination findings, is integrated into the clinical reasoning process and is used to guide treatment decisions. Unfortunately, there are inter-rater reliability and standardisation issues associated with the manual performance of this type of assessment. In an attempt to remedy these issues researchers have developed mechanical devices for the measurement of spinal stiffness. The aim of this research is to investigate the comfort and safety of a novel device for measuring P-A trunk stiffness in a sample of young adults.

**Methods:**

A sample of young adults from a general population was recruited in May 2016 from Sydney, Australia. Demographic, anthropometric and clinical variables were collected prior to participants undergoing a lumbar P-A trunk stiffness assessment involving a mechanical indentation device called the VerteTrack. The primary outcomes for the study were key feasibility items; overall assessment time, perceived comfort measured both during and after the procedure, and adverse events. Univariate ordinal logistic regression was used to identify key variables associated with a participant’s subjective report of comfort both during and after the VerteTrack assessment.

**Results:**

Eighty four participants (35% female) with a median age of 23 years (IQR = 3) took part in the research. The mean assessment time for the Vertetrack assessment was 11.6 min (SD = 2.1). Increasing load (*p* < 0.001) and increasing number of days with lower back pain (*p* = 0.009) were associated with decreased comfort ratings during the procedure. The vast majority 63/84 (75%) of participants rated the overall assessment experience as comfortable. There were two minor, short-lived adverse events recorded leading to an adverse event rate of 2.4% (2/84).

**Conclusions:**

The results of this study suggest that the VerteTrack device is well-tolerated and can be used safely and efficiently when measuring P-A stiffness of the lumbar trunk in young adults.

**Trial registration:**

Not applicable.

**Electronic supplementary material:**

The online version of this article (doi:10.1186/s12998-017-0153-z) contains supplementary material, which is available to authorized users.

## Background

Clinicians who use manual therapy use a variety of methods to examine patients presenting with spinal disorders including patient interview, special tests and physical exam. With these tools, a clinician seeks to collect and assemble all relevant clinical data for the purposes of forming a working diagnosis, prognosis and management plan for their patient [1–3].

One aspect of physical examination commonly used by clinicians is the assessment of spinal posterior to anterior (P-A) stiffness. Traditionally, P-A load/s of between 30 and 200 Newtons [4] (N) are manually applied to the spinous process of a target vertebra or group of vertebrae with the patient in a prone or seated position. The clinician then compares this subjective measurement of stiffness to those taken in adjacent areas of the patient’s spine. Simultaneously, the clinician also makes comparisons with experiential data from previous patient states/encounters as well as experiential data from their clinical training to determine ‘normal’ and ‘abnormal’ responses to the test procedure [3]. The aim of this assessment is to provoke pain and/or to determine the degree of resistance or compliance of these structures and the associated soft-tissues to loading [5]. This information, combined with other patient-specific history and examination findings, is integrated into the clinical reasoning process and is used to guide treatment decisions. The assessment of spinal P-A stiffness can also be used as a post-treatment outcome [2, 6].

Unfortunately, researchers have highlighted problems with the manual performance and interpretation of this type of examination; specifically poor inter-rater reliability [1, 7, 8], and a lack of standardisation of both the testing procedure and the scales used to quantify the stiffness ratings [1]. Nicholson et al. [5] also highlighted that low palpation sensitivity and limited subjective perception may also contribute to the poor reliability during stiffness assessments of viscoelastic structures such as the spine. Furthermore, clinicians do not use consistent forces when manually assessing spinal stiffness which can complicate the interpretation of the information being perceived [2]. This has led researchers to develop mechanical indentation devices that mimic this style of examination and provide a standardised and reliable measure of this clinical construct [9–15]. The majority of the devices described in the literature involve a motorised indenter head mounted on a gantry that applies a P-A load to a single spinal segment. Typically a series of increasing loads (measured in Newtons) are applied to the target segment, with measurement of deformation (mm) used as a proxy for movement in the target segment and the associated tissues. A modulus of stiffness (N/mm) is then calculated for that spinal level. One of the challenges associated with previous devices is that in order to measure the stiffness of multiple spinal segments (e.g. lumbar spine) the device must be repositioned and recalibrated at each segment, a cumbersome and time consuming procedure for all involved. In an attempt to streamline the automated measurement of spinal stiffness, a novel device called the Vertetrack has been developed. The device, which is currently used for research purposes, features a rolling indenter head capable of measuring the combined P-A stiffness of the spine and the adjacent tissues (trunk stiffness) without the need for repositioning and recalibration between segments/regions of the spine.

The aim of this study was to investigate the comfort and safety of the Vertetrack, a research tool for measuring P-A lumbar trunk stiffness in a sample of young adults. This information, when combined with data on accuracy and reliability, will help to determine the suitability of this device for use in research and clinical settings.

## Methods

### Design

Cross-sectional study.

### Ethics approval

Ethics approval was obtained from the Macquarie University human ethics committee – Reference number: 5201600008.

### Sampling

A convenience sample of young adults were recruited from the student population at Macquarie University, Australia. The research was conducted within a dedicated laboratory space.

### Eligibility criteria

Participants who were ≥18 years of age were eligible to participate in the study. Due to the nature of the assessment procedure, participants were excluded if they were: unable to lie in the prone position for ≥20 min; were in the second or third trimester of pregnancy; were unable to maintain their breathing cycle in passive expiration (Functional residual capacity) for at least 10 s; or had recently undergone head, neck, thoraco-abdominal or spinal surgery. The presence or absence of spinal pain was not included as part of the eligibility criteria.

### Outcome measures

All participants were issued with an information and consent form. Consenting participants then completed a baseline questionnaire which contained questions regarding demographic (age, gender, ethnicity) and clinical variables (low back pain frequency in the past 7 days, and smoking status). With reference to low back pain frequency, participants were asked *“How many days did you have low back pain during the last week?”* with the answer recorded on a scale from zero to seven. The data pertaining to smoking status was sorted into three categories: *never smoked*, *used to smoke but have now quit* (past smoker), and *current smoker*.

Upon completion of the baseline questionnaire, participants underwent a physical examination designed to capture relevant anthropometric characteristics: seated height (cm), standing height (cm), weight (kg), waist P-A diameter and circumference (cm), and chest P-A diameter and circumference (cm). Standing height and weight measurements were converted into body mass index (BMI) units (kg/m^2^). The primary outcomes for the study were: lumbar P-A trunk stiffness assessment: overall assessment time, perceived comfort, adverse events and mechanical downtime. Time was measured in minutes, and comfort level was measured on a 7-point bipolar-scale adapted from the work of Hernandez et al. [16]; 0 = strong discomfort, 1 = moderate discomfort, 2 = mild discomfort, 3 = neutral, 4 = mild comfort, 5 = moderate comfort, and 6 = strong comfort. Perceived comfort was rated by the participant both during the assessment and also at the completion of the assessment. Adverse events were recorded by the research assistant. Participants were also asked to describe their experience of the VerteTrack device at the end of the assessment.

### VerteTrack device

**Fig. 1:**
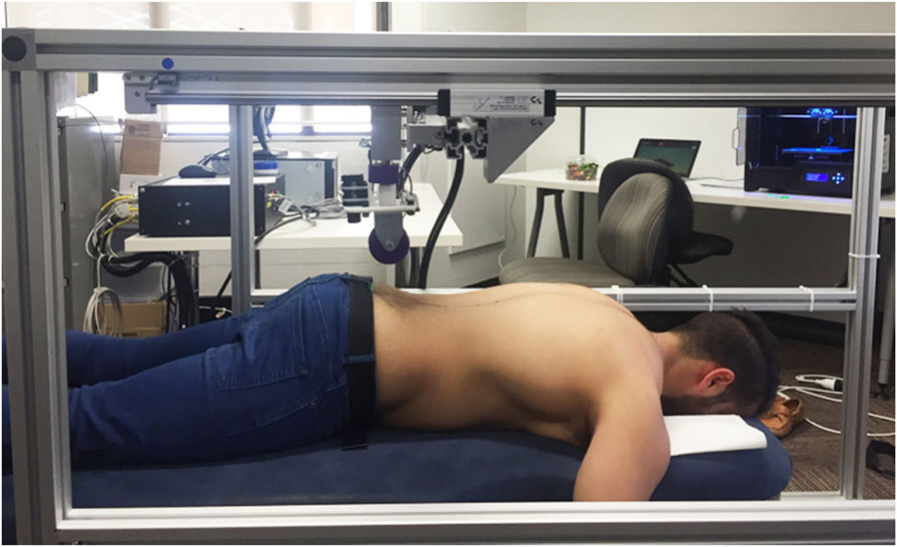
The VerteTrack device positioned over a patient on a standard plinth

**Fig. 2:**
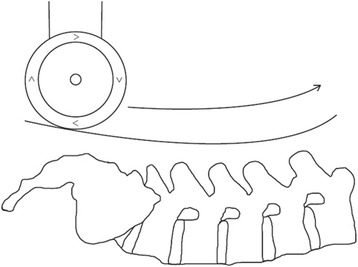
VerteTrack indentation roller

The VerteTrack device was used to measure the lumbar P-A trunk stiffness of participants. The VerteTrack device functions to apply a pre-selected vertical load continuously over a specific spinal region (e.g. lumbar spine). The device consists of a solid, cube-shaped aluminium gantry (Width 1080 mm × Height 1090 mm × Length 1510 mm), on lockable casters, that can be positioned over a participant lying in the prone position on a standard padded-plinth (Fig. 1). The frame is used to provide a rigid support for the indenter apparatus which applies a vertical load to the region of interest and houses a sensor to measure the resulting tissue deformation. The indenter apparatus consists of a rod suspended within a linear bearing to permit near-frictionless vertical translation and an indentation roller comprising two circular plastic disks (Diameter 70 mm, width 15 mm). Force transfer to the test subject is via the rod loaded with masses of increasing magnitude applied through the indentation roller. These straddle the test subject’s spinous processes thus providing a rolling contact point for the application of P-A loads (Fig. 2).

The indentation roller is moved in the X (longitudinal, superior-inferior), Y (transverse, left-right) and Z (vertical, P-A) axes by a stepping motor system (Resolution = 0.007 mm) (Stepperonline.com, China). The vertical position of the indenter relative to the frame is measured by a string potentiometer which provides real-time feedback to the control system (Resolution = 0.020 mm) (TE Connectivity, USA). Control of all motors and acquisition of the potentiometer sensor signal is controlled through custom software written in Labview (National Instruments, USA). Using this program, it is possible to position the indenter apparatus at defined way-points along the spine then have the indenter apparatus follow the pre-defined trajectory. The result is a continuous and real-time quantification of the bulk deformation of any spinal region for a given mass over a defined trajectory. Using a series of increasing masses, the force-deformation profile of the spinal region of interest can then be produced.

As the indenter roller is moved over the lumbar region, the spine and associated soft tissues are loaded by the weight of the indenter unit which can be increased or decreased by increments of 10 N (Maximum = 60 N) using weight plates. Movement of the indentation roller along a spinal region (X and Y axes) is planned by the operator who uses a mounted laser to identify way-points along the area of the trunk to be tested (Typically each spinous process). The indentation roller then follows these points over the bony and soft tissue contours of the patient’s lumbar spine, which includes the area between each way-point, through a smooth curvilinear trajectory termed the *trace*.

During data acquisition, the indentation roller is mechanically lowered onto the participant’s trunk. The Z-axis stepping motor is used only to set down then lift off the indenter roller at the beginning and end of a trace respectively. Once the indenter roller has been positioned on the subject’s trunk, there is no further contribution to the movement in the negative Z-axis from the stepping motor during a trace. Any P-A movement that is achieved is created via the effect of gravity on the mass of the indenter roller/arm and any additional weight plates. This ensures continual contact between the indenter head and the test subject’s trunk and serves to maintain a gravity-constrained rate of loading during the assessment. When testing is complete, the stepping motor system lifts the indenter roller and indenter apparatus from the subject. A video of the VerteTrack in action has been included in Additional file 1.


Additional file 1:VerteTrack being used on a model. (mov 58240 kb)


### Lumbar P-A trunk stiffness assessment

**Fig. 3:**
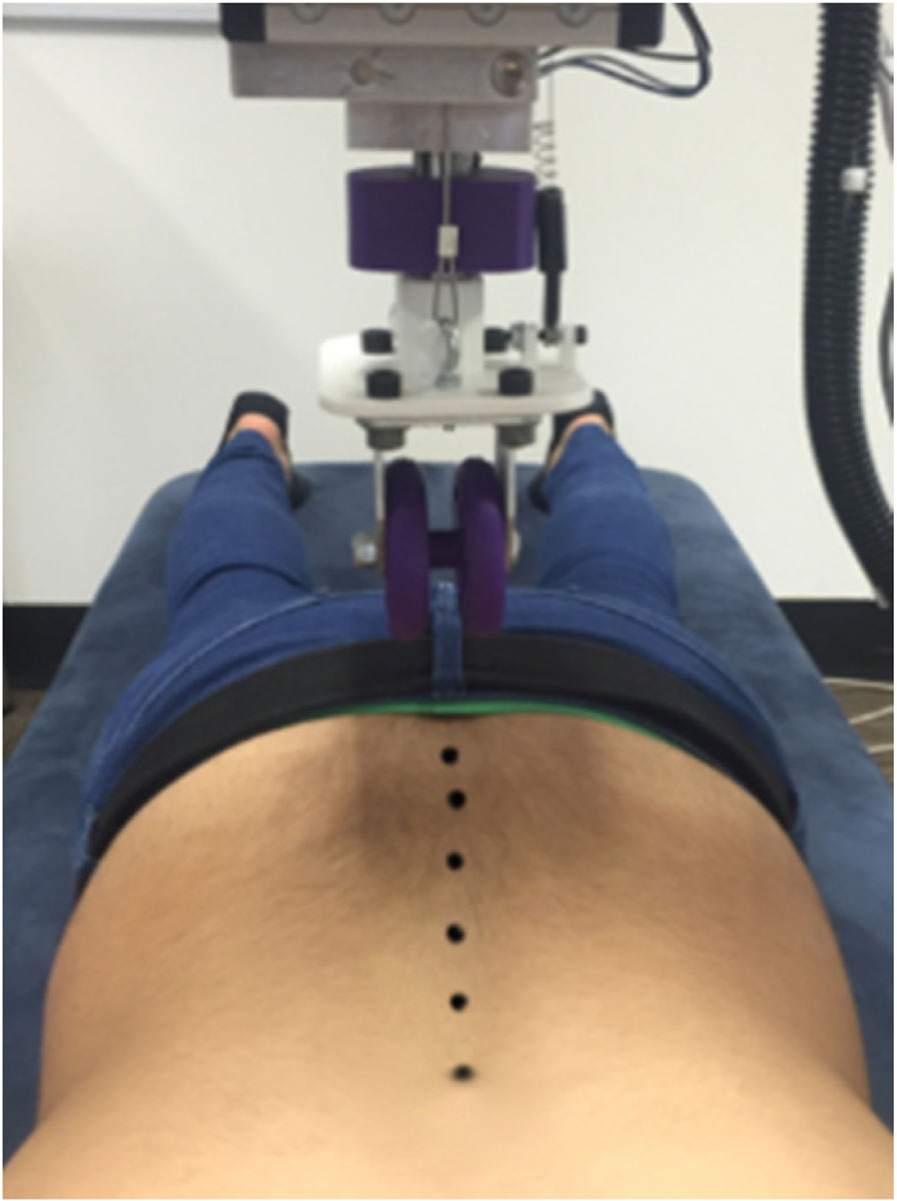
Bony surface landmarks for the creation of an individual-specific trajectory file

Participants in this study were required to wear gowns that opened at the back allowing researchers to access their spine and upper sacrum. Participants were asked to lie prone on the plinth. Once a participant was comfortable on the plinth, researchers palpated and marked the spinous processes of the sacrum (*landing point*), each of the lumbar vertebrae, and the 12th thoracic vertebrae (*lift-off point*). The Vertetrack gantry was then moved over the participant until the indentation roller was positioned over the *landing point*, which in this study was the first sacral tubercle. A vertically-orientated laser (GLX Laser Site, Barska) in line with the roller wheel apex was then turned on and then used to align the roller to each marked way-point on the subject’s back (i.e. spinous processes). When the laser matched the marks, the position was recorded and a roller trajectory calculated (Fig. 3).

Once the roller trajectory had been established, the roller was lowered onto the *landing point*. The participant was then instructed to breathe in, then passively expire (functional residual capacity [FRC]). The indenter head was then moved through the pre-calculated roller trajectory by the stepping motor system while the roller itself was free to displace the bony and soft tissues of the lumbar region in the Z-axis (~10 s). When the indenter head reached the end of the trajectory at the 12th thoracic vertebra, it was raised off the participant and returned to the region just above the landing point. The participant was then instructed to breathe normally. The initial trace (0 N) represents the minimal load configuration which includes the unweighted load receptacle, the indenter arm and the indenter roller. No additional weight is added to this initial trace which is used as the baseline measurement. Successive 10 N weights were then added to the load receptacle (up to a maximum load of 60 N) after each trace, with a rest period of approximately 1 min between loading cycles. Participants had access to an emergency stop button in the event that they felt discomfort and wished to terminate the assessment. In all, participants received seven traces with incrementally increasing loads (0 N, 10 N, 20 N, 30 N, 40 N, 50 N and 60 N). Data from the traces were compiled and used to calculate the terminal stiffness of the lumbar region. Terminal stiffness is defined as the ratio of the maximal applied force (60 N) to the maximal resultant displacement [13, 17], and was calculated for the palpated spinal levels corresponding to L5, L3 and L1. Localisation of L5 spinous process was achieved using a combination of bony landmark and motion palpation as recommended by Merz et al. [18].

### Quantitative analyses

Data were collated, cleaned, and analysed and descriptive statistics were generated for all variables. The results for the descriptive analysis are reported as mean with standard deviation (SD) or median and interquartile range (IQR). Univariate ordinal logistic regression was used to identify any variables correlated with a participant’s overall comfort rating. Univariate mixed-effects ordinal logistic regression was then performed to identify independent variables that were correlated with a participant’s comfort rating during each loading phase. The mixed-effects model includes a random effect for participant to control for the repeated measures on each participant. The results for the ordinal logistic regression analyses are reported as odds ratios with 95% confidence intervals and *p*-values. The significance level (α) was set at 0.05, and all assumptions were checked and determined to have been met unless otherwise reported. All analyses were conducted using the statistical software package R, version 3.1.2 (R Core Team, Vienna, Austria).

### Qualitative analyses

All mechanical and adverse events were described in full. Content analysis [19] was used to determine the major and minor themes in the data relating to the participant’s experience of the VerteTrack assessment.

## Results

Eighty four participants (35% female) consented to participate in the research. The age data were right skewed with a median age of 23 years (IQR = 3). With reference to ethnicity, 57% (48/84) were Caucasian, 33% (28/84) were Asian, 1% (1/84) were Pacific Islander and 7% (7/84) were not stated. The descriptive statistics for anthropometric variables are detailed in Table 1.

**Table 1:** Descriptive statistics for the anthropometric variables

Variable	Mean	Median	Standard Deviation	Interquartile Range	Range (Min, Max)
Sitting height (cm)	171.6	172	9.5	15.2	145.0, 190.0
Standing height (cm)	90.8	91	5.4	8.5	77.0, 102.0
Weight (kg)	72.0	69.4	15.8	19.1	44.1, 124.2
BMI (kg/m^2^)	24.3	23.8	3.9	4.1	17.4, 37.0
Waist circumference (cm)	84.2	82.5	10.4	11.6	66.0, 118.0
Chest circumference (cm)	94.5	93.0	9.6	9.5	78.0, 127.0
Waist P-A diameter (cm)	18.7	18.0	3.4	4.5	12.5, 28.5
Chest P-A diameter (cm)	18.5	18.5	3.2	4.0	12.5, 27.5

*P-A* posterior to anterior, *cm* centimetres, *kg* kilograms, *m* ^*2*^ metres squared, *min* minimum, *max* maximum

**Fig. 4:**
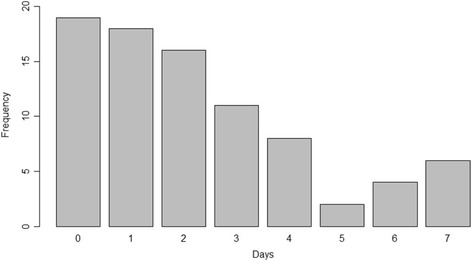
Barchart of self-reported days with lower back pain in the past week

**Fig. 5:**
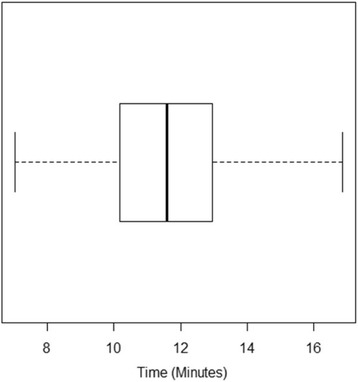
Boxplot of trunk stiffness assessment times

With regards to smoking status, 82% (69/84) of participants reported that they had never smoked, 15% (13/84) reported that they used to smoke but have now quit, and 2% (2/84) of the sample were current smokers. A large proportion (77.3%, [65/84]) of the sample population reported one or more days of lower back pain in the week prior to the assessment (Fig. 4). There were several outliers in the assessment time data (Fig. 5) which represented cases where software or equipment did not function as expected (Equipment events). The time taken to reboot software and/or recalibrate the device contributed to these outliers which have been removed from the analysis. Assessment times were normally distributed with a mean assessment time of 11.6 min with a standard deviation of 2.1 min (Fig. 5).

**Fig. 6:**
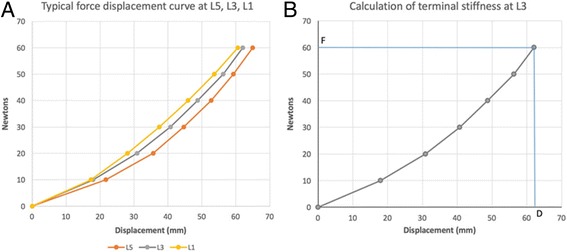
**a** Force Displacement Curve. Typical force-displacement curves for levels L5, L3 and L1 (Participant #43). **b** Calculation of Terminal Stiffness. Terminal stiffness is calculated by dividing the maximum applied force (F) by the maximal displacement (D)

In an attempt to standardize the stiffness measurements between the VerteTrack and the other devices described in the literature, terminal stiffness measures were calculated for three points in lumbar region - L5, L3, and L1 (Fig. 6A & B). The mean terminal stiffness at the level of L5 was 1.0 N/mm (SD = 0.15), and at the level of L3 was 1.0 N/mm (SD = 0.16). The terminal stiffness data for L1 were right skewed with a median of 1.0 N/mm (IQR = 0.21). All participants received the full 60 N loading to the lumbar region.

**Fig. 7:**
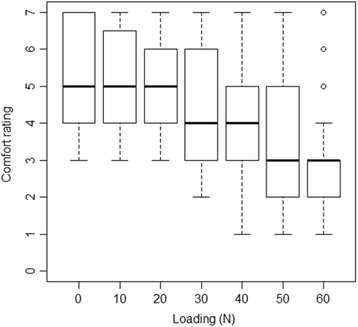
Boxplots of comfort ratings with incremental loading **°** = Outlier

The comfort ratings for the cohort are shown in Fig 7. With respect to overall comfort, the most frequent rating was that of *neutral* or *mild comfort*. There were no observations seen for the *strong comfort* or *strong discomfort* categories in the overall comfort data. Collapsing the overall comfort data into two categories (comfortable or uncomfortable) highlighted that 75% of the sample population rated the overall experience as comfortable.

### Univariate analyses

Mixed-effects ordinal logistic regression was used to model the influence of each of the variables age, gender, assessment time, number of days with lower back pain in the past week, BMI, smoking status and load in Newtons on participant comfort ratings during each loading phase. Of these variables, the presence of an increasing number of days with lower back pain in the past week, and increasing load were significantly associated with decreased comfort ratings (Table 2). Ordinal logistic regression was used to model the influence of these same variables (Excluding individual loads) on the overall comfort rating for the assessment. There was no evidence to suggest that any of these variables were associated with the overall comfort rating.

**Table 2:** Results from the univariate analyses of the comfort data

	Overall comfort (ordinal logit model)		Comfort during the assessment (mixed-effects ordinal logit model)	
Variable [*Reference Category*]	Odds ratio (95% CI)	*p*-value	Odds ratio (95% CI)	*p*-value
Age	1.06 (0.96, 1.18)	0.235	1.00 (0.90, 1.12)	0.975
Assessment time (minutes)	1.00 (0.99, 1.00)	0.772	1.00 (0.92, 1.09)	0.939
BMI (kg/m^2^)	0.96 (0.87, 1.05)	0.377	1.04 (0.94, 1.16)	0.417
Gender [*Female*] - Male				
	1.44 (0.63, 3.32)	0.386	0.98 (0.41, 2.36)	0.970
Low back pain frequency	0.85 (0.70, 1.02)	0.103	0.77 (0.64, 0.94)	0.009*
Load (Newtons)			0.90 (0.89, 0.91)	<0.001*
Smoking Status [*Never smoked*]				
- *Current smoker*	0.15 (0.02, 1.48)	0.087	0.11 (0.01, 1.63)	0.108
- *Past smoker*	0.86 (0.32, 2.31)	0.762	0.83 (0.27, 2.56)	0.740

* = significant, *BMI* Body mass index, *kg* kilograms, *m* metres

### Equipment events

There were four unexpected equipment-related events. On four occasions, software or equipment did not function as expected during the trunk stiffness assessment. These events required the research assistant to either reboot the software and/or reposition the device before continuing the assessment. No ill-effects were reported by the participants in these cases.

### Adverse events

There were two adverse events reported. One participant reported an aggravation of pre-existing rib pain in the day following the stiffness assessment. Another participant reported aggravation of pre-existing lower back pain resulting in bilateral referred leg pain during the assessment. Of the two participants that made these reports, the reactions were short-lived and neither required remedial treatment. Therefore, the rate of adverse events using the VerteTrack device in this population was 2.4% (2/84). Although some participants reported some discomfort during the stiffness assessment, the emergency stop button was not utilised at any time during this study.

### Participant experience

Content analysis was performed by a single author (BTB) on the data relating to the participant’s subjective experience of the VerteTrack assessment. Comments were first sorted into three categories; *positive*, *negative* and *mixed*. Comments that contained both positive and negative remarks, or neutral statements were categorised as *mixed*. Eighty two participants provided feedback on the VerteTrack assessment. Purely *positive* comments were made by 29% (24/82) of the sample, 45% (37/82) gave *mixed* responses and 24% (20/82) made *negative* comment. Participants who made *positive* comment typically stated that the VerteTrack assessment was comfortable/therapeutic and enjoyed the novel experience. Major themes in the *mixed* and *negative* categories were that the indenter roller was perceived as being too rough and/or hard which created a pinching or catching sensation as it rolled up the upper lumbar spine. Participants also commonly reported discomfort as load was increased, particularly in the upper lumbar spine. Minor themes included reports that being in the prone position for the assessment was somewhat uncomfortable, the indenter head reproduced pain, and that the style of the assessment was unusual/foreign.

## Discussion

The aim of this study was to investigate the comfort and safety of the VerteTrack for measuring P-A lumbar trunk stiffness in a sample of young adults.

With regard to safety, the rate of adverse events associated with the use of the VerteTrack device in this population was low (2.4%). The adverse events that did occur were thought to be related to Vertetrack testing, but this could not be confirmed with the study design. Adverse events attributed to Vertetrack testing were minor and short-lived, and involved two participants with pre-existing neuromusculoskeletal conditions.

There is a paucity of data with respect to the safety (specifically harms data) relating to other mechanical indentation devices in the literature. The majority of the work in this area has been conducted on young, asymptomatic populations where adverse events are seldom discussed or reported. Edmondston et al. [12] investigated the effect of body position on the spinal stiffness at L3 and L5 in 12 asymptomatic participants with an average age of 28.8 years. The mechanical device utilised by Edmondston et al. applied loads of between 30 and 80 N, and was equipped with a panic button facility. The researchers stated that no participant had cause to activate the panic button at any time during the research. However, Edmondston et al. also listed *“undue pain or discomfort during testing and development of any adverse symptoms that may have been associated with the procedures”* as a withdrawal criteria in their methods section. It is therefore unclear whether or not there were participants who were excluded from the final analysis due to adverse reactions. In addition, Latimer et al. [15] developed a mechanical device for measuring spinal stiffness that was capable of delivering force up to 105 N to a test subject. These researchers reported that since developing their device over 100 test subjects had underwent an assessment with only one of these subjects reporting a minor, short-duration adverse event.

Based on an analysis of the comfort data, both during and after the procedure, it appears that the VerteTrack assessment was considered comfortable by the majority (75%) of participants in this study. A strong theme that emerged however in both the qualitative and quantitative analyses was that comfort ratings were inversely related to loading i.e. increasing loads resulted in lower comfort ratings. Kumar and Stoll [11] state that loads between 45 and 135 N allow for reliable measurement of spinal stiffness while still being comfortable. Based on the descriptive statistics, participants found that comfort began to decrease with loading ≥30 N, which is considerably lower than the values suggested by Kumar and Stoll. It may be the case that the rolling indenter head is less tolerable at higher loads when compared to indenter heads that move in a single plane only. In addition, participants who had experienced one or more days of lower back pain in the past week (77.3% of the sample) were also more likely to give a lower comfort rating compared to asymptomatic individuals. Therefore, while participants may report discomfort with increasing load or a history of back pain, we could not determine if the discomfort was from the test itself, was from provocation of sub-clinical conditions or was due to excessive loading. As tolerance for this type of testing appears to be individual in nature, we recommend that testing be performed to the participant’s onset of discomfort rather than an absolute loading value.

The nature of the sensation produced by the rolling indenter head on the trunk was an important factor governing a participant’s subjective experience of the assessment. This rolling action of the VerteTrack is novel when compared to the other mechanised tools for measuring spinal stiffness. The two wheels that form the rolling indenter head were 3D printed and made from Acrylonitrile butadiene styrene (ABS) plastic making them rigid and inflexible. Other non-rolling devices described in the literature have employed padded indenter heads [9–12, 20]. It is likely that using a different material and/or providing some form of padding or lubricant for the device-subject interface may improve comfort. Any padding that is employed however, has the potential to contribute to measurement error through compression/deformation. It is therefore important that accuracy is not sacrificed in the quest for comfort. In their study, Lee and Evans [10] employed talcum powder in an attempt to reduce friction between the indenter head and the skin of the participants. In this research the indenter head was programmed to roll in a caudal to cephalad direction which may have caused discomfort and/or skin irritation as the indenter head moved ‘uphill’ from the apex of the lumbar lordosis to the *lift-off* point in the thoracolumbar junction. As the VerteTrack device can also be programmed to move in a cephalad to caudal direction it would be worthwhile establishing which direction or combination of directions is ideal for testing.

With regards to the time taken to perform the assessment, this research highlights that the lumbar trunk stiffness of a young adult can be assessed efficiently (mean assessment time of 11.6 min) using the VerteTrack device. Allison et al. [21] investigated the influence of varied load orientation on lumbar spine stiffness in 24 normal subjects using the spinal P-A mobilization (SPAM) apparatus. These researchers reported that it took approximately 30 min to perform 12 measurements of the lumbar spine (L5, L3 and L1) for each participant at loads between 30 and 100 N. Lee and Evans [10] measured the influence of loading rate on spinal stiffness at L3, L4 and L5 in a group of young adults. These authors reported that their assessment, including varied loading rates at three segment levels, took approximately 2 h to complete. While other researchers [9, 11–13] have utilised similar mechanical indentation devices for measuring spinal stiffness there is lack of data on the overall time taken to perform these assessments. It is important to note that the assessment time data in this study were skewed by a number of instances of software failure. The version of the software used in this study represented version 2.0. Future iterations of the software associated with the VerteTrack will lead to fewer software bugs and therefore shorter assessment times. Still, the time taken to test participants in this study highlights a significant improvement from prior devices, making the VerteTrack device suitable for use in clinical trials.

There are several limitations associated with this study. Firstly, the spinal stiffness assessments were performed on a convenience sample made up of young adults (median age 23 years, range = 18–38 years) from a tertiary-level student population. It is not clear how safe, comfortable or efficient this device would be for use in a paediatric or geriatric population. Furthermore, the sample was comprised of people from a general (non-clinical) population, which given the high prevalence of back pain in society included participants with and without varying levels of low back discomfort. The suitability of the VerteTrack device remains unclear in clinical research or clinical practice settings.

Interestingly, there was a higher than expected prevalence of LBP in our sample population. This may have been due to the fact that sample consisted entirely of chiropractic students studying in the postgraduate and undergraduate programs at Macquarie University. It is known that chiropractic students suffer from physical side-effects, e.g. lower back pain, from performing [22, 23] and/or receiving [24] spinal manipulative procedures during their training which may have contributed to acute cases of lower back pain in the sample. Participation in the study was entirely voluntary, however it is possible that the students that elected to participate were those who were either experiencing an acute low back pain episode or had a history of lower back pain. With the spine being a strong focus in chiropractic education, students in the program may demonstrate a hypersensitivity to spinal dysfunction which may have skewed the prevalence figures for lower back pain in this sample.

The terminal stiffness values and the variation between the various spinal levels obtained in this study are smaller than those observed in previous studies. Due to the action of the indenter roller it is likely that the displacement values obtained with the VerteTrack differ when compared to other devices as a function of time under load. The indenter roller moves quickly over the trunk and applies P-A loads up to a maximum of 60 N. Both the rate of loading and magnitude of loading are unique in this study making comparisons between the terminal stiffness values obtained by previous devices and the VerteTrack problematic. This highlights the need to better understand the nuances involved with the interpretation of the trunk stiffness values obtained with this device.

Harms data were collected actively at the time of the trunk stiffness assessment and there was no planned active or passive harms surveillance post-assessment. Latent adverse reactions may have been captured more thoroughly via the inclusion of a dedicated follow-up period, but attribution of these events to testing is difficult.

In this study, there was a negative correlation between comfort ratings and loading. Loads were applied sequentially to the lumbar spine of a participant with a brief rest period between traces. A more robust determination of the comfort associated with the assessment may have been obtained by using a Latin square design, similar to the work by Edgecombe et al. [25], to assign loads in a random sequence.

As this form of assessment provides clinical data that is used alongside other diagnostic information to guide management decisions, it is of crucial importance that the reliability and accuracy of any mechanical indentation device be tested. Further research should also be performed in a variety of trunk regions in clinical populations of varying ages to determine the true value of the VerteTrack device in these scenarios.

## Conclusions

Based on the findings of this study, it appears that the VerteTrack is well-tolerated by participants and can be used safely and efficiently to measure the P-A stiffness of the lower trunk in young adults. The device is strictly for research purposes at this time, however the ultimate suitability of the VerteTrack for use in research and clinical settings may be determined once data from this study is combined with data regarding the reliability and accuracy of the device.

## Abbreviations

BMI: Body mass index
cm: Centimetres
FRC: Functional residual capacity
kg: Kilograms
mm: Millimetres
N: Newtons
N/mm: Newtons per millimetre
P-A: Posterior to anterior
SS: Spinal stiffness
